# Effects of SmartStax^®^ and SmartStax^®^ PRO maize on western corn rootworm (*Diabrotica virgifera virgifera* LeConte) larval feeding injury and adult life history parameters

**DOI:** 10.1371/journal.pone.0288372

**Published:** 2023-07-10

**Authors:** Jordan D. Reinders, William J. Moar, Graham P. Head, Safeer Hassan, Lance J. Meinke

**Affiliations:** 1 Department of Entomology, University of Nebraska, Lincoln, Nebraska, United States of America; 2 CropScience Division, Bayer AG, Chesterfield, Missouri, United States of America; Assam Agricultural University Faculty of Agriculture, INDIA

## Abstract

Field-evolved resistance of the western corn rootworm (WCR), *Diabrotica virgifera virgifera* LeConte, to *Bacillus thuringiensis* Berliner (Bt) proteins Cry3Bb1 and Cry34/35Ab1 (now classified as Gpp34Ab1/Tpp35Ab1) expressed in the pyramid SmartStax^®^ has been documented in areas of the United States (U.S.) Corn Belt. SmartStax^®^ PRO is a recently registered rootworm-active pyramid containing the same Bt proteins expressed in SmartStax^®^ plus DvSnf7 dsRNA. Little to no published data is available comparing efficacy of the technologies or potential effects of dietary exposure on adult WCR fitness. Therefore, experiments were conducted to compare effects of adult WCR dietary exposure to SmartStax^®^ and SmartStax^®^ PRO on life history parameters and efficacy of the technologies in the field with both Bt-susceptible and Bt-resistant WCR populations. WCR life history parameters evaluated included adult longevity, head capsule width, egg production, and egg viability. Results of small-plot field trials indicated that both technologies provided a high level of root protection when a Bt-susceptible WCR population was present. Root protection was reduced on SmartStax^®^ but maintained on SmartStax^®^ PRO when WCR Bt resistance occurred. Lifetime egg production was the key life history parameter that was significantly reduced when either Bt-susceptible or Bt-resistant adult WCR were fed SmartStax^®^ or SmartStax^®^ PRO diet. A potential fitness advantage was apparent as egg production was significantly higher in the Bt-resistant than Bt-susceptible population. The similar response by the Bt-susceptible WCR population to SmartStax^®^ and SmartStax^®^ PRO indicates that results were caused by sublethal dietary exposure to Bt proteins. Adult size (males < females) and egg viability (high: >95%) were not significantly different among treatments but longevity results were inconsistent between years. Collectively, the field efficacy and life history parameter data expand existing knowledge of SmartStax^®^ and SmartStax^®^ PRO technologies, which will inform practical WCR resistance management programs.

## Introduction

The western corn rootworm (WCR; *Diabrotica virgifera virgifera* LeConte, Coleoptera: Chrysomelidae) is an important insect pest of maize (*Zea mays* L.) throughout the United States (U.S.) Corn Belt [1, 2]. Larval feeding can cause significant economic damage by decreasing water and nutrient uptake, gas exchange, and plant growth [3–5], potentially reducing grain yield by 15–17% [6, 7] for each node of root injury [8]. The WCR has a long history of adaptation to management strategies in various parts of the U.S. Corn Belt, including field-evolved resistance to crop rotation [9], some formulated products from four insecticide classes [reviewed in 10], and maize hybrids expressing *Bacillus thuringiensis* Berliner (Bt) proteins [reviewed in 11]. In areas of continuous maize production (i.e., ≥ 2 consecutive growing seasons), build-up of WCR densities and resistance evolution contribute to annual management challenges [2, 12, 13].

The use of rootworm-active Bt hybrids has largely replaced insecticide applications as the primary WCR management strategy in continuous maize since commercialization in the early-mid 2000s [10, 14]. Three Bt proteins were initially registered and commercialized as single-trait events in transgenic maize hybrids: Cry3Bb1 in 2003 [15], Cry34/35Ab1 (now reclassified as Gpp34/Tpp35Ab1 [16]) in 2005 [17], and mCry3A in 2006 [18]. The high-dose refuge-based resistance management strategy has been a key component of mandated U.S. Environmental Protection Agency (U.S. EPA) insect resistance management (IRM) policy when Bt maize is deployed in the field [19]. When high-dose expression (i.e., 25x the dose needed to kill 99.99% of susceptible individuals [20]) of a Bt protein occurs, resistance evolution has often been prevented or delayed [21, 22]. A U.S. Corn Belt example is the European corn borer, *Ostrinia nubilalis* (*Hübner*), which has not evolved practical resistance [as defined by 23] in the field after widespread planting of Bt hybrids expressing Cry proteins (e.g., Cry1Ab, Cry1F) commercialized since 1996 [24, 25]. The high-dose refuge strategy failed when applied to rootworm-Bt maize because key assumptions of the strategy were violated [14]. The lack of *in planta* high-dose expression of rootworm-Bt proteins [26–28] was a key factor that limited corn rootworm IRM. As a result, continuous cultivation of single-protein maize hybrids led to WCR field-evolved resistance in areas of the U.S. Corn Belt over time [11, 13, 29–37]. Evidence of cross-resistance among the Cry3 proteins (Cry3Bb1, mCry3A, and eCry3.1Ab) and a lack of cross-resistance between the Cry3 proteins and Cry34/35Ab1 have also been well-documented [29, 31, 38, 39].

To improve resistance management, maize hybrids expressing single Bt proteins were replaced in recent years by pyramided hybrids that express two rootworm-active Bt proteins [37, 40]. Current rootworm-active Bt pyramids include: Cry3Bb1 + Cry34/35Ab1 [41], mCry3A + Cry34/35Ab1 [42], and mCry3A + eCry3.1Ab [43]. In initial field tests, high efficacy was obtained when the rootworm-Bt pyramid containing Cry3Bb1 + Cry34/35Ab1 (SmartStax^®^) was evaluated [44, 45], but the long-term use and increasing frequency of WCR resistance to Cry3Bb1 [36, 37, 46] and Cry34/35Ab1 [13, 32, 36, 47] have reduced the potential IRM value of Bt pyramids in some Corn Belt locations. The confirmation of WCR field-evolved resistance to Bt pyramids containing a Cry3 protein and Cry34/35Ab1 documented in Iowa [36] and Nebraska [37] raises concerns over future Bt protein efficacy and underscores the need for new products with novel modes of action targeting the WCR.

SmartStax^®^ PRO is a rootworm-active transgenic pyramid containing the two commercialized Bt proteins expressed in SmartStax^®^ (Cry3Bb1, Cry34/35Ab1) and the new DvSnf7 dsRNA construct [48], constituting the first corn rootworm product containing three modes of action. The *Snf7* (sucrose non-fermenting 7) ortholog identified in WCR, *DvSnf7* [49], acts as part of the ESCRT-III pathway responsible for internalizing, transporting, sorting, and degrading transmembrane proteins [50, 51]. Ingestion of DvSnf7 dsRNA triggers activation of the WCR RNA interference (RNAi) machinery, leading to suppression of DvSnf7 mRNA and eventual larval mortality [51, 52]. A lack of cross-resistance between Cry3Bb1 and DvSnf7 dsRNA has been documented [53–55], confirming that SmartStax^®^ PRO contains three different modes of action.

To date, some negative effects on WCR life history parameters have been recorded after dietary exposure to individual Bt proteins in SmartStax^®^ and SmartStax^®^ PRO (e.g., Cry3Bb1, Cry34/35Ab1 [47, 56–61] but little information is available when WCR ingest DvSnf7 dsRNA or Bt pyramid tissue. Comparative data are also lacking when WCR–Bt pyramid interactions take place in a Bt-resistant versus Bt-susceptible population. Results of initial field trials indicated that SmartStax^®^ PRO significantly reduced WCR root injury [62] and adult emergence [62, 63] in several areas of the Corn Belt with suspected or confirmed Bt resistance, suggesting the inclusion of DvSnf7 dsRNA may provide significant IRM value to the pyramid. In addition to larval mortality, SmartStax^®^ PRO negatively affects various adult life history parameters. Previous research documented significant sublethal effects of adult dietary exposure to SmartStax^®^ PRO ear tissue on adult longevity and lifetime egg production in a WCR population from Colfax County, Nebraska, with confirmed resistance to SmartStax^®^ [63]. These results suggested one or more components of this pyramid contributed to the negative effect on WCR life history parameters and population growth. However, this experiment did not include comparisons between SmartStax^®^ and SmartStax^®^ PRO or between Bt-susceptible and Bt-resistant WCR populations. To date, no research on WCR fitness after dietary exposure to SmartStax^®^ has been published.

Therefore, the first objective of this study was to utilize small-plot trials to compare WCR larval feeding injury on SmartStax^®^, SmartStax^®^ PRO, and non-rootworm Bt (‘non-RW Bt’) maize hybrids in fields with either Bt-susceptible or Bt-resistant WCR populations. The second objective was to compare three adult WCR diet treatments (i.e., SmartStax^®^, SmartStax^®^ PRO, and non-RW Bt maize) for potential effects on WCR life history parameters of Bt-susceptible and Bt-resistant WCR populations. Collectively, the life history parameter and field efficacy data will expand the existing knowledge base regarding these two rootworm technologies and inform practical IPM and IRM programs.

## Materials and methods

### Field performance of SmartStax^®^ and SmartStax^®^ PRO

Three on-farm research sites located in eastern Nebraska were used to conduct small-plot field trials in 2022 (Table 1). A site with Bt-susceptible WCR was located at the University of Nebraska Eastern Nebraska Research, Extension, and Education Center (ENREEC) near Ithaca in Saunders County (WCR population: ‘Saunders’). WCR collected from this site have been consistently susceptible to Bt proteins in plant-based bioassays conducted in previous studies [13, 31, 34, 37]. Annual small-plot research is conducted at this site with a large surrounding area planted to non-Bt maize to maintain Bt susceptibility. Two commercial maize fields were also selected with WCR populations exhibiting some level of resistance to multiple Bt proteins confirmed in past plant-based bioassays [13, 37]. One Bt-resistant site, located in Stanton County (WCR population: ‘Stanton’), has been planted with rootworm-Bt maize pyramids containing a Cry3 protein and Cry34/35Ab1 from 2015 to 2022. A second Bt-resistant site was a maize field in Cuming County (WCR population: ‘Cuming 1’) characterized by cultivation of rootworm-Bt pyramids containing a Cry3 protein and Cry34/35Ab1 from 2016 to 2022.

**Table 1:** Planting and root evaluation information for field trials conducted in Saunders, Stanton, and Cuming counties, 2022.

Trial	Planting Date ^a^	Hybrid Maturity ^b^	Root Evaluation Date ^c^
Saunders	10 May 2022	110RM, 112RM	18 July 2022
Stanton	11 May 2022	110RM	26 July 2022
Cuming 1	16 May 2022	112RM	27 July 2022

^a^Four replications of non-rootworm Bt, SmartStax^®^, and SmartStax^®^ PRO maize were planted at each site. Individual plots were four rows wide by 6m in length replicated in a randomized complete block design.

^b^Two hybrid maturity dates were used in this study. Both maturity dates were planted at the Saunders Co. location.

^c^Ten maize plants were excavated from each plot and evaluated using the 0–3 node-injury scale according to Oleson et al. [8].

Field plots were established with pure stands of non-RW Bt, SmartStax^®^, and SmartStax^®^ PRO maize hybrids from similar genetic backgrounds measuring four rows wide (76cm row spacing) by 6m long. Treatments were replicated four times in a randomized complete block design. A seeding rate of 80,000 seeds per hectare was planted using a Kinze® 2100 four-row planter (planting dates in Table 1). A 110-day relative maturity hybrid was planted in Saunders and Stanton counties and a 112-day relative maturity hybrid was planted in Saunders and Cuming counties (Table 1). All plots were kept free of volunteer maize plants and weeds throughout the growing season. In mid to late July, ten maize plants were excavated from the center two rows of each field plot (40 plants per treatment) at all trial locations and the 0–3 node-injury scale [8] was used to evaluate larval feeding injury on each maize plant (Table 1).

### Single-plant bioassay technique

During the 2021 growing season, a minimum of 150 gravid females were collected from each trial site to obtain progeny for use in single-plant bioassays (developed by Gassmann et al. [29]) conducted in 2022 to confirm the susceptibility of each WCR population to SmartStax^®^. In addition, four diapausing WCR colonies initially collected in the 1990s and maintained by the USDA-ARS North Central Agricultural Research Laboratory in Brookings, South Dakota, were assayed and pooled into a composite susceptible control sample for comparison to the field populations. These WCR colonies were collected prior to the commercialization of rootworm-active Bt proteins in 2003 and have been continuously reared without the addition of wild-type genes. Neonate F_1_ progeny were assayed on two maize hybrids without seed treatments: 1) DKC 66–87 GENVT2P (non-RW Bt) and 2) DKC 64–34 GENSS (SmartStax^®^). The general bioassay methodology used at the University of Nebraska has been outlined in Wangila et al. [31] and Reinders et al. [34]. In brief, 12 maize plants of each hybrid were grown in 1L plastic containers (Johnson Paper & Supply Company, Minneapolis, MN) to the V5 growth stage [64]. Plants were infested with 12 neonate WCR larvae (≤24h after eclosion) and maintained in growth chambers set at 24°C with a 14h:10h (L:D) photoperiod. After larval feeding for 17d, survivors from each maize plant were collected in a jar containing 70% ethanol below a Berlese funnel.

### Life history parameter experiment

Experiments were conducted in 2021 and 2022 with WCR populations from UNL-ENREEC in Saunders County (WCR population: ‘Saunders’) and a commercial maize field in Cuming County (WCR population: ‘Cuming 2’) to characterize the effects of adult exposure to SmartStax^®^, SmartStax^®^ PRO, and non-RW Bt maize ear tissue (i.e., husk, silk, kernels, and cob; hereafter ‘diet’ or ‘adult diet’) on Bt-susceptible and Bt-resistant WCR life history parameters. Saunders and Cuming 2 populations were susceptible or exhibited a level of resistance to Bt proteins, respectively, in past plant-based bioassays [13, 37]. The commercial maize field in Cuming County from which the Cuming 2 population was collected was planted with single-trait Cry34/35Ab1 hybrids from 2016 to 2018 and a Cry3 + Cry34/35Ab1 pyramid from 2019 to 2021. The Saunders County site was managed as previously described to maintain WCR susceptibility to Bt proteins. To confirm WCR susceptibility to SmartStax^®^, plant-based bioassays were conducted as previously described on F_1_ progeny from both WCR populations each year the life history parameter experiments were performed.

Approximately 500 WCR adult females were collected from each field site during August 2020 and 2021. WCR were transported to the University of Nebraska-Lincoln and maintained in 28cm^3^ plexiglass cages under specified laboratory conditions for egg collection (F_1_ progeny) and storage during diapause [31, 34]. After diapause termination in the spring following adult collection, post-diapause development and egg hatch was facilitated by placing WCR eggs at 25°C until larval eclosion. Neonate F_1_ progeny were reared to adulthood following the standard University of Nebraska rearing protocol [Appendix II in 65] to obtain adults for use in the life history parameter experiments. Emerging adults from each WCR population were placed in separate 28cm^3^ plexiglass cages by date. Less than 24h post-emergence, one male and one female WCR were subsequently placed into polystyrene oviposition boxes (5.9cm × 5.9cm × 7.8cm; ShowMan Box, Althor Products, Windsor Locks, CT) with a 2.5cm cross-section of adult diet placed inside of a rectangular plastic shelf (4.5cm × 2.5cm × 1.5cm) attached to the lid of each box with Velcro® [66]. A moistened oviposition substrate (65g silty clay loam soil, 20mL distilled water) was added to each box as an oviposition site. In 2021 experiments, 60 male/female pairs were established per maize hybrid and 50 male/female pairs were established per maize hybrid in 2022 experiments.

Pre-commercial non-RW Bt, SmartStax^®^, and SmartStax^®^ PRO maize hybrids were grown in a University of Nebraska-Lincoln greenhouse according to the methods outlined in Reinders et al. [63] to obtain maize ear tissue for the life history parameter experiments conducted in 2021 and 2022. In brief, maize seeds of each hybrid were grown to the R2-R3 stage [64] in individual raised wooden planter boxes (2m × 2m × 1m) filled with native silty clay loam soil. Expression of Cry3Bb1 and Cry34Ab1 was confirmed at the V5 growth stage using QuickStix lateral flow strips (Envirologix, Inc., Portland, ME). Cry3Bb1 and DvSnf7 dsRNA are linked on the same T-DNA insertion; therefore, positive expression of Cry3Bb1 confirms expression of DvSnf7 dsRNA in SmartStax^®^ PRO plants [53]. Tassel bags (Seedburo Equipment Company, Des Plaines, IL) were used to prevent pollen shed and each ear was hand-pollinated to prevent cross-pollination between maize hybrids. Adult diet was replaced every 3-4d to ensure diet quality and protein expression were maintained.

Life history parameters measured in this experiment included adult longevity, adult head capsule width, lifetime egg production, and egg viability. Adult longevity was calculated as the difference between the date of laboratory emergence and the date of mortality in oviposition boxes. Mortality was recorded when adult diet was replaced every 3-4d. The head capsule width of dead beetles was measured to the nearest 0.01mm with an AmScope 3.5X-90X Simul-Focal Trinocular Stereo Zoom microscope with attached 18MP USB3 Camera (United Scope LLC, Irvine, CA). Lifetime egg production was determined by washing the contents of individual oviposition boxes through a U.S.A. Standard Testing Sieve No. 60 (Thermo Fisher Scientific, Waltham, MA) to remove soil. WCR eggs were subsequently washed onto a milk filter (KenAg, Ashland, OH) and counted using a stereo microscope. After counting, eggs from each WCR population × adult diet combination were combined and stored in Petri dishes (Thermo Fisher Scientific, Waltham, MA) according to the methods outlined in Reinders et al. [63] until the following spring at which time egg viability was determined. Fifty random eggs from each WCR population × adult diet combination were transferred to a Petri dish containing a moistened Whatman™ Qualitative Filter Paper: Grade 1 Circle (Thermo Fisher Scientific, Waltham, MA). This process was replicated for each WCR population × adult diet combination based on the number of eggs available (total n = 12 or 18 Petri dishes per combination). Hatching neonates were counted and removed from Petri dishes twice daily to calculate the proportion of viable eggs.

### Data analysis

SAS 9.4 software [67] was used to analyze all data and statistical significance was reported at α = 0.05. Separate analyses were conducted within each maize hybrid maturity date (110RM, 112RM) in field trials. Analyses were also conducted separately for each life history parameter assessed (head capsule width, longevity, egg production, egg viability) within each year a life history parameter experiment was conducted (2021, 2022).

### Field performance of SmartStax^®^ and SmartStax^®^ PRO

The field performance of each maize hybrid was assessed based on root damage due to larval feeding (node injury score, [8]). Individual maize plants were given a node injury score following a continuous distribution within the restricted positive interval, 0–3. Mean root damage ratings from each plot were divided by three to calculate proportional root injury (0–1 scale) and were analyzed using a beta-binomial distribution [68]. A beta-binomial distribution was chosen because the data are restricted by a positive, continuous interval (0–3) that does not extend to ± ∞ (Gaussian distribution) [69]. Field performance (i.e., node injury ratings) was evaluated using a generalized linear mixed model (GLMM; GLIMMIX procedure [67]). Trial location, maize hybrid, and their interaction were included in the model as fixed factors. Field replication nested within trial location was included in the model as a random factor. Mean root damage ratings and associated standard errors reported in this manuscript are from the trial location × maize hybrid interaction LSMEANS table.

### Life history parameter analysis

A GLMM (GLIMMIX procedure [67]) was used to analyze the effect WCR population, beetle sex, and adult diet on adult longevity with data fit to a negative binomial distribution. Model fixed factors included WCR population, beetle sex, adult diet treatment, and their interactions. Oviposition box nested within the WCR population × beetle sex × adult diet interaction was included in the model as a random factor. The LSMEANS statement with the SLICEDIFF option was used to identify significant differences in mean adult longevity among model factors. Tukey’s HSD test was used to control for type I error rates when making multiple comparisons. The effect of WCR population, beetle sex, and adult diet on adult head capsule width was analyzed using a linear mixed model (LMM; GLIMMIX procedure [67]) with data fit to a normal distribution. The remainder of the adult head capsule width analysis follows the procedure outlined previously. Adult longevity and adult head capsule width means and associated standard errors reported in this manuscript are from the WCR population × beetle sex × adult diet interaction LSMEANS table.

Similarly, a GLMM (GLIMMIX procedure [67]) was used to analyze the effect of WCR population and adult diet on lifetime egg production (data fit to negative binomial distribution) and the proportion of viable eggs (data fit to beta distribution). WCR population, adult diet, and the WCR population × adult diet interaction were included in the model as fixed factors. Oviposition box nested within the WCR population × adult diet interaction was included in the model as a random factor. The LSMEANS statement with the SLICEDIFF option was used to identify significant differences in life history parameters among model factors. Tukey’s HSD test was used to control for type I error rates when making multiple comparisons. Lifetime egg production and egg viability means and associated standard errors reported in this manuscript are from the WCR population × adult diet interaction LSMEANS table.

### Single-plant bioassay analysis

For each population, bioassay proportional survival on non-RW Bt and SmartStax^®^ maize was calculated by dividing the number of larval survivors recovered from each Berlese funnel sample by 12. Corrected survival on SmartStax^®^ maize was calculated as the complement of corrected mortality using Abbott’s correction [70] and was defined as survival on each SmartStax^®^ plant divided by mean survival on non-RW Bt maize for each population [36, 37]. A generalized linear model (GLM; GLIMMIX procedure [67]) following a normal distribution was used to evaluate corrected survival on SmartStax^®^ maize among WCR populations. The model included WCR population as a fixed factor. The DIFF option was used to identify significant differences in SmartStax^®^ corrected survival between WCR populations. Bioassay results from WCR life history experiment populations (2021, 2022) and field performance populations (2022) were analyzed separately.

## Results

### Field performance of SmartStax^®^ and SmartStax^®^ PRO

**Fig 1:**
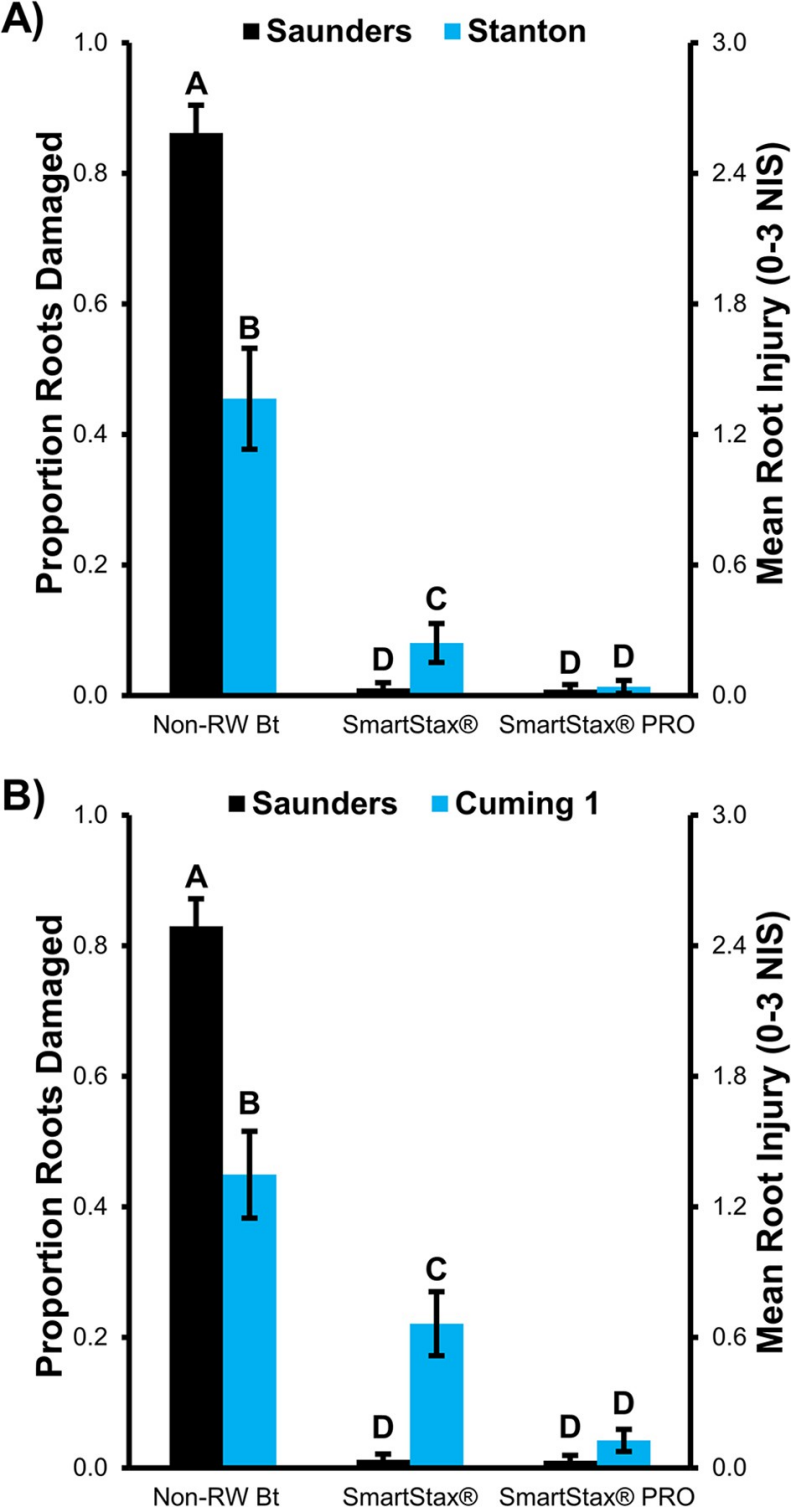
Field performance of non-rootworm Bt, SmartStax^®^, and SmartStax^®^ PRO maize at three northeast Nebraska trial locations, 2022. A) Mean root injury (± SE) of 110RM maize planted at trial locations in Saunders and Stanton counties, B) Mean root injury (± SE) of 112RM maize planted at trial locations in Saunders and Cuming counties. The Saunders population was susceptible to SmartStax^**®**^ and the Stanton and Cuming 1 populations exhibited moderate and high levels of resistance to SmartStax^**®**^, respectively. Ten maize plants were excavated from each plot (total N = 40 per treatment) and evaluated using the 0–3 node injury scale [8]. Bars with the same letter are not significantly different (GLMM, *P*>0.05).

A significant trial location by maize hybrid interaction effect on root injury was observed in the 110RM maize trials grown in Saunders and Stanton counties (*F*_2,12_ = 11.98, *P* = 0.0014) and the 112RM maize trials grown in Saunders and Cuming counties (*F*_2,12_ = 29.54, *P*<0.0001). Similar trends were observed in the 110RM and 112RM trials. Mean rootworm injury on non-RW Bt maize was significantly greater at the Saunders location than either the Stanton or Cuming 1 location; mean root injury on non-RW Bt maize was similar at the Stanton and Cuming 1 locations. At the Saunders location (Bt-susceptible), root injury was very low and not significantly different between SmartStax^®^ and SmartStax^®^ PRO treatments (Fig 1). In contrast, mean root injury on SmartStax^®^ maize was significantly lower than mean root injury on non-RW Bt maize but significantly greater than injury on SmartStax^®^ PRO maize at both locations with Bt-resistant WCR (Fig 1).

### Life history parameter experiment

**Fig 2:**
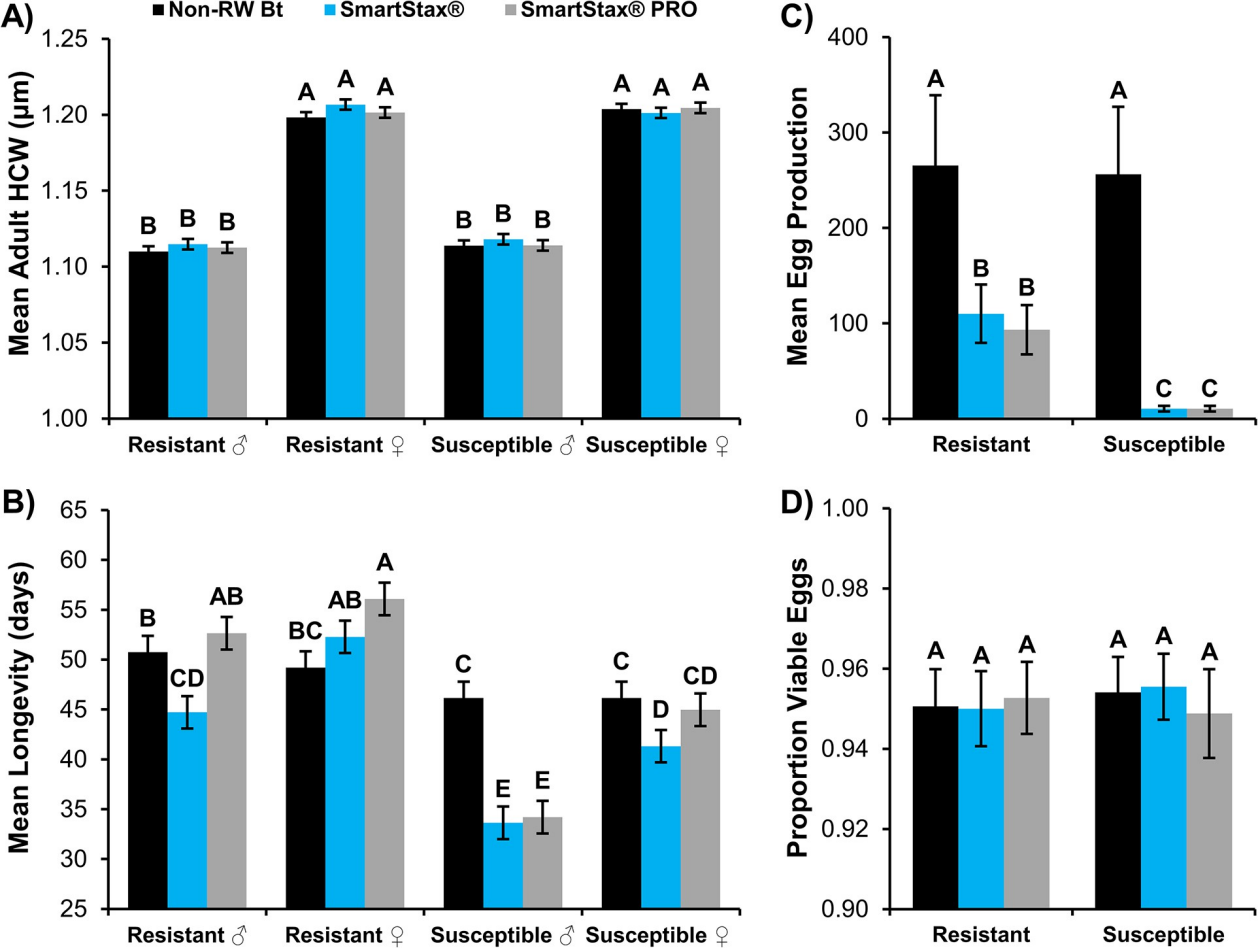
Life history parameters characterized during adult exposure experiments, 2021. A) Mean (± SE) adult head capsule width (HCW; mm), B) Mean (± SE) adult longevity (days; d), C) Mean (± SE) egg production, and D) Proportion (± SE) of viable F_1_ eggs. A total of 60 male/female pairs were established for each adult diet. Bars with the same letter are not significantly different (GLMM or LMM, *P*>0.05).

**Fig 3:**
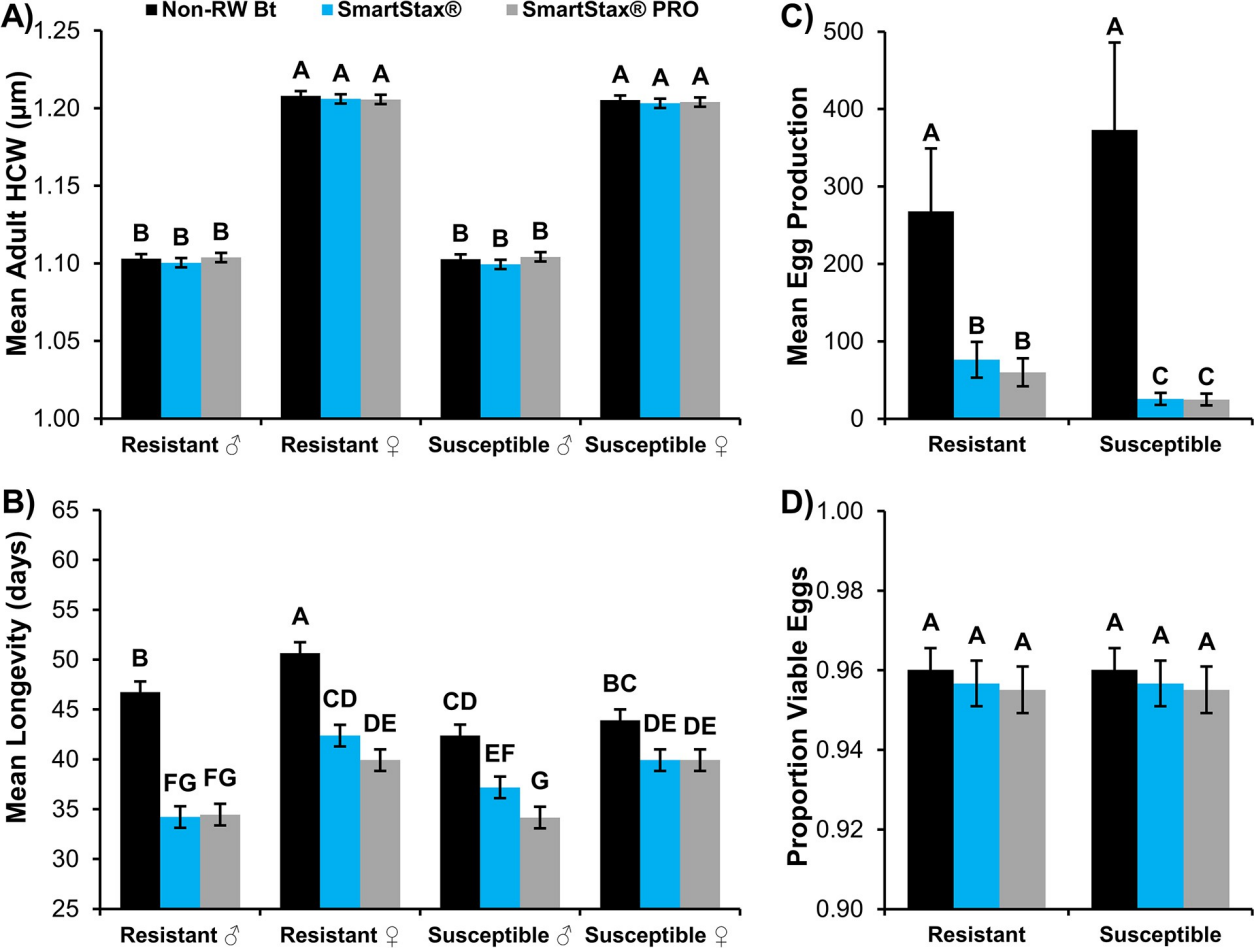
Life history parameters characterized during adult exposure experiments, 2022. A) Mean (± SE) adult head capsule width (HCW; mm), B) Mean (± SE) adult longevity (days; d), C) Mean (± SE) egg production, and D) Proportion (± SE) of viable F_1_ eggs. A total of 50 male/female pairs were established for each adult diet. Bars with the same letter are not significantly different (GLMM or LMM, *P*>0.05).

Mean adult WCR head capsule width was significantly affected by beetle sex in 2021 (*F*_1,708_ = 1990.32, *P*<0.0001) and 2022 (*F*_1,588_ = 3523.65, *P*<0.0001) life history parameter experiments (Figs 2A and 3A). All other model effects and their interactions were not significant (S1 Table). The head capsule width of adult female WCR, regardless of population or adult diet, was approximately 0.09mm greater than their male counterparts in both years (Figs 2A and 3A).

The effects and interaction of WCR population, adult diet, and beetle sex on mean adult WCR longevity were more variable between experimental years (S1 Table). The WCR population × adult diet interaction (*F*_2,708_ = 11.66, *P*<0.0001) and the adult diet × beetle sex interaction (*F*_2,708_ = 8.33, *P* = 0.0003) significantly affected mean adult longevity in 2021 experiments (S1 Table). Mean longevity of Bt-resistant males and females was significantly greater than Bt-susceptible males and females, respectively, when fed SmartStax^®^ or SmartStax^®^ PRO ear tissue (Fig 2B). Mean Bt-resistant male longevity was also significantly longer on non-RW Bt maize than longevity of Bt-susceptible males, but this trend was not apparent for females (Fig 2B). In contrast, mean longevity of Bt-susceptible male WCR fed non-RW Bt maize was significantly greater than mean longevity of Bt-susceptible males fed SmartStax^®^ or SmartStax^®^ PRO maize (Fig 2B). A similar trend was observed for the Bt-susceptible female comparison of mean longevity on non-RW Bt versus SmartStax^®^ diet but was not apparent when non-RW Bt and SmartStax^®^ PRO diet treatments were compared (Fig 2B). Bt-susceptible females lived approximately 7.5d and 11d longer than their male counterparts when fed SmartStax^®^ and SmartStax^®^ PRO maize, respectively (Fig 2B).

In 2022 experiments, the WCR population × adult diet interaction (*F*_2,588_ = 8.90, *P* = 0.0002) and the WCR population × beetle sex interaction (*F*_1,588_ = 4.01, *P* = 0.0457) significantly affected mean adult longevity (S1 Table). The highest longevity within each WCR population × beetle sex combination was documented when adults were fed non-RW Bt maize; mean longevity was significantly reduced when adults were fed SmartStax^®^ and SmartStax^®^ PRO maize (Fig 3B). Within the Bt-resistant WCR population, female longevity was significantly longer than male longevity when fed non-RW Bt, SmartStax^®^, or SmartStax^®^ PRO ear tissue (Fig 3B). This trend was only apparent in the Bt-susceptible population when males and females were fed SmartStax^®^ PRO diet (Fig 3B). While Bt-resistant males lived significantly longer than Bt-susceptible males fed non-RW Bt maize, there were no significant differences in mean longevity between Bt-resistant and Bt-susceptible males when fed SmartStax^®^ or SmartStax^®^ PRO maize; this trend was also observed between Bt-resistant and Bt-susceptible females (Fig 3B).

Mean egg production was significantly affected by the interaction of WCR population and adult diet in both experimental years (2021: *F*_2,354_ = 30.16, *P*<0.0001; 2022: *F*_2,294_ = 3.23, *P* = 0.0410; S2 Table). Overall, no significant difference in egg production was observed between WCR populations exposed to the non-RW Bt maize diet (Figs 2C and 3C). A significant decrease in mean egg production was observed when WCR were fed an adult diet of SmartStax^®^ or SmartStax^®^ PRO maize; however, this decrease was less pronounced in the Bt-resistant WCR population (Figs 2C and 3C). Each year, the Bt-susceptible WCR population exhibited a 93–96% reduction in mean egg production when exposed to an adult diet of SmartStax^®^ or SmartStax^®^ PRO relative to egg production on non-RW Bt maize. In contrast, the significant reduction in mean egg production of Bt-resistant WCR was only 59–72% and 65–78% on SmartStax^®^ and SmartStax^®^ PRO diets, respectively (Figs 2C and 3C). The proportion of viable F_1_ eggs was not significantly affected by WCR population, adult diet, or their interaction in 2021 or 2022 experiments (S2 Table), indicating that egg viability was not significantly different among treatments (Figs 2D and 3D). Egg hatch was relatively high (≥ 95%) in both experimental years, indicating most eggs laid were viable.

### Single-plant bioassays

#### Field performance of SmartStax^®^ and SmartStax^®^ PRO WCR populations

The main effect WCR population significantly affected corrected survival on SmartStax^®^ maize (*F*_3,36.22_ = 44.24, *P*<0.0001). No significant difference in corrected survival on SmartStax^®^ maize was observed between the susceptible control and the Saunders population (Table 2). Both the Stanton and Cuming 1 WCR populations had significantly greater corrected survival relative to the susceptible control and Saunders population. However, the highest SmartStax^®^ corrected survival was documented in the Cuming 1 population (Table 2).

**Table 2:** Mean corrected survival (±) SE of western corn rootworm populations from field performance experiments assayed on SmartStax^®^ maize using the Gassmann single-plant technique, 2022. Means within a column followed by the same letter are not significantly different (GLM, *P* > 0.05).

WCR Population	SmartStax^®^ Corrected Survival^1^
Susceptible Control (USDA-ARS NCARL)	0.011 (0.007)a
Saunders (Bt-susceptible)	0.047 (0.043)a
Stanton (Bt-resistant)	0.564 (0.063)b
Cuming 1 (Bt-resistant)	0.845 (0.110)c

^1^Corrected survival = $\frac{{Survival\;on\;SmartStax}^{®}\;}{Survival\;on\;non-rootworm\;Bt}$

#### Life history parameter experiment WCR populations

In 2021 bioassays, the main effect WCR population significantly affected corrected survival on SmartStax^®^ maize (*F*_2,14.61_ = 35.75, *P*<0.0001). The susceptible control exhibited the lowest corrected survival on SmartStax^®^ maize (Table 3). Both the Saunders and Cuming 2 WCR populations had significantly greater corrected survival relative to the susceptible control. However, significantly greater corrected survival on SmartStax^®^ maize was documented in the Cuming 2 population compared to the Saunders population (Table 3). In bioassays conducted in 2022, the main effect WCR population also significantly affected SmartStax^®^ corrected survival (*F*_2,15.17_ = 26.90, *P*<0.0001). The susceptible control and Saunders population exhibited very low corrected survival on SmartStax^®^ maize (Table 3). Similar to 2021 bioassays, the Cuming 2 WCR population exhibited the highest corrected survival, indicating a high proportion of resistant individuals within this population (Table 3).

**Table 3:** Mean corrected survival (±) SE of western corn rootworm populations used in life history parameter experiments assayed on SmartStax^®^ maize using the Gassmann single-plant technique, 2021 and 2022. Means within a column followed by the same letter are not significantly different (GLM, *P* > 0.05).

	Corrected Survival^1^ on SmartStax^®^	
Field Site	2021 Bioassays	2022 Bioassays
Susceptible Control (USDA-ARS NCARL)	0.014 (0.006)a	0.011 (0.006)A
Saunders (Bt-susceptible)	0.127 (0.030)b	0.047 (0.020)A
Cuming 2 (Bt-resistant)	0.627 (0.077)c	0.500 (0.066)B

^1^Corrected survival = $\frac{{Survival\;on\;SmartStax}^{®}\;}{Survival\;on\;non-rootworm\;Bt}$

## Discussion

The performance of SmartStax^®^ PRO was consistent across the 110RM and 112RM trials when either Bt-susceptible or Bt-resistant WCR populations were present. The large reduction in WCR larval feeding injury on SmartStax^®^ PRO compared to the non-RW Bt observed at the Bt-resistant trial locations (Stanton: 90.6%; Cuming 1: 97.1%) and reductions at the Bt-susceptible site (Saunders: 98.5–99.0%) bear this out (Fig 1). This high level of WCR control relative to non-RW Bt maize was also apparent when SmartStax^®^ was planted at the susceptible site (Saunders: injury reduction of 98.5–98.7%) A similar result was obtained with SmartStax^®^ (injury reduction of 98.1–99.0%) in a 2021 trial conducted at a different Bt-susceptible site in Saunders County [71]. In contrast, the level of root injury increased on SmartStax^®^ relative to the non-RW Bt hybrid in a Bt-resistant environment, providing lower root protection (injury reduction, Stanton: 82.3%; Cuming 1: 50.9%). Only moderate root injury (mean node injury scale [NIS; 0–3 scale] rating of 1.35) in the non-RW Bt treatment was recorded at each Bt-resistant trial location, but this level of rootworm pressure was enough to cause a mean injury rating in the Cuming 1 SmartStax^®^ treatment (NIS = 0.67) that exceeded the established unexpected injury threshold of 0.5 for pyramided hybrids [14, 72]. The difference in SmartStax^®^ resistance levels between the Cuming 1 and Stanton sites confirmed in plant-based bioassays (Table 2) may explain why the Stanton mean root rating on SmartStax^®^ remained below the unexpected injury level threshold.

It is noteworthy that the relative difference in control between SmartStax^®^ PRO and SmartStax^®^ remained fairly constant (SmartStax^®^ PRO / SmartStax^®^ NIS; Stanton: 0.16; Cuming 1: 0.19) under different Bt resistance scenarios. This was the result of increased root injury ratings in both treatments as the level of resistance increased (Fig 1). It would be interesting to determine if the root injury level would continue to rise in SmartStax^®^ and SmartStax^®^ PRO treatments at similar rates at the two Bt-resistant locations under increased rootworm pressure (e.g., to the high-level present at the Saunders location). Both Stanton and Cuming 1 populations exhibited a significant increase in corrected survival in SmartStax^®^ bioassays relative to the susceptible control and a higher level of root injury compared to non-RW Bt maize, indicating this is an example of practical resistance. Data from this study demonstrate that WCR density and Bt resistance must be at certain levels to cause greater than expected injury and lead to practical resistance in the field. It is unknown if the level of root injury recorded at Cuming 1 could lead to significant yield loss since grain yield was not recorded in this study. Field results from this study provide additional evidence that inclusion of DvSnf7 dsRNA in SmartStax^®^ PRO can enhance root protection when WCR resistance to SmartStax^®^ is present. Additional data is needed to evaluate the impact of the Bt resistance level × rootworm density interaction on maize root injury and yield when either SmartStax^®^ or SmartStax^®^ PRO is deployed in the field, especially when WCR densities are high.

The most significant WCR life history parameter affected by SmartStax^®^ and SmartStax^®^ PRO in adult dietary exposure experiments was lifetime egg production (Figs 2 and 3). The large reduction in lifetime egg production after dietary exposure to SmartStax^®^ or SmartStax^®^ PRO was similar among the two treatments and consistent within Bt-susceptible or Bt-resistant populations each year. Of note was the significant increase in egg production by the Bt-resistant population compared to the Bt-susceptible population when fed SmartStax^®^ or SmartStax^®^ PRO diet (Figs 2C and 3C). This suggests an increase in reproductive fitness occurred when a level of Bt resistance was present in the WCR population. A positive correlation between adult female size and fecundity [73] and a direct relationship between adult male size and mating potential have been observed [74], which can contribute to differences in egg production. However, the lack of significant differences in male or female size among adult diet treatments in this study (Figs 2A and 3A) implicates the roles of sublethal exposure to the pyramid technologies and Bt resistance as the key factors influencing egg production. Because egg viability remained very high (≥95%) across all adult diets in both Bt-resistant and Bt-susceptible populations (Figs 2D and 3D), data suggest that even though large reductions in egg production can occur after dietary exposure to SmartStax^®^ or SmartStax^®^ PRO, most eggs that are laid will be viable. This trend was consistent with results from previous Bt-resistant WCR dietary exposure experiments with SmartStax^®^ PRO [63].

With the exception of Bt-susceptible females fed an adult diet of SmartStax^®^ PRO maize in the 2021 experiment, Bt-susceptible WCR fed an adult diet of SmartStax^®^ or SmartStax^®^ PRO maize exhibited decreased adult longevity compared to non-RW Bt maize (Figs 2B and 3B). Reduced longevity has also been reported in susceptible WCR populations exposed to sublethal doses of Cry3Bb1 [56] and Cry34/35Ab1 [60]. Bt-resistant WCR adult female longevity was inconsistent between experimental years, with similar or increased longevity associated with SmartStax^®^ and SmartStax^®^ PRO exposure in 2021 and significantly decreased longevity after exposure to both pyramids in 2022 (Figs 2B and 3B). The 2022 data is consistent with past SmartStax^®^ PRO dietary experiments with a WCR Bt-resistant population [63]. The reasoning for the inconsistent results in longevity among years in the Bt-resistant WCR population is unclear. Separate adult WCR collections were made in 2020 and 2021 from different areas of the commercial maize field from which Cuming 2 was obtained, which resulted in a slightly higher level of SmartStax^®^ resistance confirmed in 2021 versus 2022 bioassays (Table 3). This may have shifted the relative ratio of resistant:susceptible individuals present in the population enough to facilitate a relative fitness advantage when exposed to the transgenic pyramids. A recent study documented a significant positive relationship between larval WCR corrected survival on Cry3Bb1 and mean larval development metrics [46]. Results indicated that as the level of WCR resistance to Cry3Bb1 increased, mean head capsule width and larval fresh weight also increased. This results in similar mean larval development time on Cry3Bb1 or non-RW Bt maize in highly resistant WCR populations [36, 75] compared to longer mean larval development and adult emergence periods often recorded when susceptible WCR populations receive sublethal dietary exposure to Cry3Bb1 maize [26, 76–79]. A similar phenomenon may occur with longevity as WCR resistance to Bt proteins increases; future research is needed to test this hypothesis.

The WCR is a protandrous species [80] and males reach sexual maturity 5-7d post-emergence [81]. Males mate with teneral females after reaching sexual maturity, and most additional mating attempts occur within the next 20d [82]. The pre-ovipositional period in WCR females can range from 6d to 21d [83]. Peak oviposition generally occurs 10-15d into the oviposition period, with oviposition occurring for up to 60d [83–85]. Since male longevity after adult exposure to SmartStax^®^ and SmartStax^®^ PRO maize was ≥34d in both Bt-susceptible and Bt-resistant populations, male mating potential will likely be unaffected under field conditions. The increased longevity observed in Bt-resistant WCR females exposed to SmartStax^®^ PRO in 2021 may increase the oviposition period, leading to a higher population density the following growing season. In contrast, the differences in adult female longevity observed in 2022 suggest that the peak oviposition period will remain largely unaffected by SmartStax^®^ and SmartStax^®^ PRO exposure (mean longevity ≥40d). However, the decreased lifespan may have contributed to the reduction in lifetime egg production evident in 2022 versus 2021 life history parameter experiments (Figs 2C and 3C).

The great similarity in negative SmartStax^®^ and SmartStax^®^ PRO treatment effects on susceptible WCR lifetime egg production and longevity (except for 2022 male longevity) suggests that sublethal dietary exposure to Cry3Bb1 and/or Cry34/35Ab1 impacted the life history parameters measured in this study. Even though DvSnf7 dsRNA does not appear to contribute to significant reductions in longevity and egg production, sublethal dietary exposure of WCR larvae and adults to RNAi technology will occur in maize fields where SmartStax^®^ PRO is planted because DvSnf7 dsRNA is expressed in below- and above-ground maize tissues [52, 86, 87]. Larval or adult exposure to DvSnf7 dsRNA in maize tissues could theoretically affect other aspects of WCR physiology [88]. It would be especially important to understand how lifetime and adult exposure over successive generations (e.g., continuous maize production systems) may affect larval susceptibility to appropriately characterize the risk of resistance evolution [88].

In conclusion, results from this study indicate that both SmartStax^®^ and SmartStax^®^ PRO provide a high level of root protection when a WCR population is susceptible to Bt proteins. The high level of root protection was reduced on SmartStax^®^ maize but maintained on SmartStax^®^ PRO maize when WCR resistance to Cry3Bb1 and Cry34/35Ab1 was present. Lifetime egg production was the key life history parameter that was significantly reduced when either Bt-susceptible or Bt-resistant WCR were fed SmartStax^®^ and SmartStax^®^ PRO diet. However, in the Bt-resistant population, a potential fitness advantage was apparent as egg production was significantly higher than that recorded in the Bt-susceptible population. The similar response by the Bt-susceptible population to SmartStax^®^ and SmartStax^®^ PRO indicates that results were caused by sublethal dietary exposure to Cry3Bb1 and Cry34/35Ab1. Results suggest that SmartStax^®^ PRO will be a useful tool to mitigate WCR Bt resistance by greatly reducing WCR densities and fecundity of survivors. However, these characteristics will also place high selection pressure on DvSnf7 dsRNA in areas with existing Bt resistance and mask ongoing Bt resistance issues in WCR populations produced in areas of continuous maize production. A laboratory selection experiment conducted by Khajuria et al. [54] documented resistance to DvSnf7 dsRNA after seven generations of selection in a field derived WCR colony, indicating natural WCR populations possess some frequency of dsRNA resistance alleles. Therefore, modeling exercises are needed to determine how SmartStax^®^ PRO dietary exposure may impact WCR population growth parameters and the potential for resistance evolution. Continuous cultivation of SmartStax^®^ PRO maize in areas with Bt resistance may erode SmartStax^®^ PRO efficacy over time and promote resistance evolution to the technology. Because the WCR is highly adaptable to management tactics [9; reviewed in 10, 11], deploying SmartStax^®^ PRO within an integrated pest management framework [14, 89, 90] is recommended to reduce selection and slow WCR resistance evolution to RNAi technology.

## Supporting information

S1 Table Tests of fixed effects from generalized linear mixed model (degrees of freedom, df; F value statistic, *F*; p-value, *P*) to evaluate effects of population, adult diet, beetle sex, and interactions on adult head capsule width and adult longevity, 2021 and 2022. (DOCX)

S2 Table Tests of fixed effects from generalized linear mixed model (degrees of freedom, df; F value statistic, *F*; p-value, *P*) to evaluate effects of population, adult diet, and the population × adult diet interaction on lifetime egg production and egg viability, 2021 and 2022. (DOCX)
