# Supplementary material for: Effects of SmartStax® and SmartStax® PRO maize on western corn rootworm (Diabrotica virgifera virgifera LeConte) larval feeding injury and adult life history parameters
Source: PLoS One. 2023 Jul 10;18(7):e0288372. doi: 10.1371/journal.pone.0288372 (PMC10332594; doi:10.1371/journal.pone.0288372)
Supplement: S1 Table — (DOCX) [file pone.0288372.s001.docx]

**S1 Table. Tests of fixed effects from generalized linear mixed model (degrees of freedom, df; F value statistic, *F*; p-value, *P*) to evaluate effects of population, adult diet, beetle sex, and interactions on adult head capsule width and adult longevity, 2021 and 2022.**

|  |  | Life History Parameter | | | | | |
| --- | --- | --- | --- | --- | --- | --- | --- |
| Year | **Model Effect** | Adult Head Capsule Width | | | Adult Longevity | | |
|  |  | df | *F* | *P* | df | *F* | *P* |
| 2021 | Population | 1, 708 | 0.93 | 0.3356 | 1, 708 | 110.07 | **<0.0001** |
|  | Adult Diet | 2, 708 | 1.19 | 0.3056 | 2, 708 | 10.74 | **<0.0001** |
|  | Beetle Sex | 1, 708 | 1990.32 | **<0.0001** | 1, 708 | 24.39 | **<0.0001** |
|  | Population × Adult Diet | 2, 708 | 0.72 | 0.4854 | 2, 708 | 11.66 | **<0.0001** |
|  | Population × Beetle Sex | 1, 708 | 0.21 | 0.6451 | 1, 708 | 2.54 | 0.1116 |
|  | Adult Diet × Beetle Sex | 2, 708 | 0.11 | 0.8954 | 2, 708 | 8.33 | **0.0003** |
|  | Population × Adult Diet × Beetle Sex | 2, 708 | 0.74 | 0.4786 | 2, 708 | 1.37 | 0.2542 |
| 2022 | Population | 1, 588 | 0.59 | 0.4428 | 1, 588 | 8.39 | **0.0039** |
|  | Adult Diet | 2, 588 | 0.81 | 0.4451 | 2, 588 | 76.99 | **<0.0001** |
|  | Beetle Sex | 1, 588 | 3523.65 | **<0.0001** | 1, 588 | 53.85 | **<0.0001** |
|  | Population × Adult Diet | 2, 588 | 0.05 | 0.9522 | 2, 588 | 8.90 | **0.0002** |
|  | Population × Beetle Sex | 1, 588 | 0.38 | 0.5392 | 1, 588 | 4.01 | **0.0457** |
|  | Adult Diet × Beetle Sex | 2, 588 | 0.45 | 0.6355 | 2, 588 | 2.24 | 0.1069 |
|  | Population × Adult Diet × Beetle Sex | 2, 588 | 0.00 | 0.9952 | 2, 588 | 1.74 | 0.1762 |

Significant effects (*P* < 0.05) are shown in bold.
