# Supplementary material for: Effects of SmartStax® and SmartStax® PRO maize on western corn rootworm (Diabrotica virgifera virgifera LeConte) larval feeding injury and adult life history parameters
Source: PLoS One. 2023 Jul 10;18(7):e0288372. doi: 10.1371/journal.pone.0288372 (PMC10332594; doi:10.1371/journal.pone.0288372)
Supplement: S2 Table — (DOCX) [file pone.0288372.s002.docx]

**S2 Table. Tests of fixed effects from generalized linear mixed model (degrees of freedom, df; F value statistic, F; p-value, P) to evaluate effects of population, adult diet, and the population** × **adult diet interaction on lifetime egg production and egg viability, 2021 and 2022.**

|  |  | Model Effect | | | | | | | | |
| --- | --- | --- | --- | --- | --- | --- | --- | --- | --- | --- |
| Year | Variable | Population | | | Adult Diet | | | Population × Adult Diet | | |
|  |  | df | *F* | *P* | df | *F* | *P* | df | *F* | *P* |
| 2021 | Lifetime Egg Production | 1, 354 | 92.99 | **<0.0001** | 2, 354 | 54.01 | **<0.0001** | 2, 354 | 30.16 | **<0.0001** |
|  | Egg Viability | 1, 96 | 0.05 | 0.8162 | 2, 96 | 0.02 | 0.9754 | 2, 96 | 0.14 | 0.8680 |
| 2022 | Lifetime Egg Production | 1, 294 | 4.92 | **0.0273** | 2, 294 | 30.34 | **<0.0001** | 2, 294 | 3.23 | **0.0410** |
|  | Egg Viability | 1, 66 | 0.00 | 1.0000 | 2, 66 | 0.46 | 0.6354 | 2, 66 | 0.00 | 1.0000 |

Significant effects (*P* < 0.05) are shown in bold.
